# Downregulation of connexin 43-based gap junctions underlies propofol-induced excessive relaxation in hypertensive vascular smooth muscle cells

**DOI:** 10.1186/s12964-023-01176-3

**Published:** 2023-06-28

**Authors:** Weiqi Zeng, Zhizhao Deng, Yingxin Gao, Guoliang Sun, Xianlong Li, Dongdong Yuan

**Affiliations:** Department of Anesthesiology, Third Affiliated Hospital of Sun Yat-Sen University, Guangzhou, 510630 Guangdong China

**Keywords:** Angiotensin II, Propofol, Gap junction, Connexin 43

## Abstract

**Background:**

Postinduction hypotension caused by propofol remains a non-negligible problem for anesthesiologists, and is especially severe in chronic hypertensive patients with long-term vasoconstriction and decreased vascular elasticity. The functional change in gap junctions composed of Cx43 (Cx43-GJs) is reported as the biological basis of synchronized contraction or relaxation of blood vessels. Thus, we investigated the role of Cx43-GJs in propofol-induced dramatic blood pressure fluctuations in chronic hypertensive patients, and their internal mechanisms.

**Methods:**

Human umbilical artery smooth muscle cells (HUASMCs) were pretreated with long-term angiotensin II (Ang II), with or without propofol, to simulate the contraction and relaxation of normal and hypertensive VSMCs during anesthesia induction. The levels of F-actin polymerization and MLC2 phosphorylation were used as indicators to observe the contraction and relaxation of HUASMCs. Different specific activators, inhibitors and siRNAs were used to explore the role of Cx43-GJs and Ca^2+^ as well as the RhoA/ LIMK2/cofilin and RhoA/MLCK signaling pathways in the contraction and relaxation of normal and hypertensive HUASMCs.

**Results:**

Both F-actin polymerization and MLC2 phosphorylation were significantly enhanced in Ang II-pretreated HUASMCs, along with higher expression of Cx43 protein and stronger function of Cx43-GJs than in normal HUASMCs. However, with propofol administration, similar to Gap26 and Cx43-siRNA, the function of Cx43-GJs in Ang II-pretreated HUASMCs was inhibited compared with that in normal HUASMCs, accompanied by a larger decrease in intracellular Ca^2+^ and the RhoA/LIMK2/cofilin and RhoA/MLCK signaling pathways. Eventually F-actin polymerization and MLC2 phosphorylation were more dramatically decreased. However, these effects could be reversed by RA with enhanced Cx43-GJ function.

**Conclusion:**

Long-term exposure to Ang II significantly enhanced the expression of the Cx43 protein and function of Cx43-GJs in HUASMCs, resulting in the accumulation of intracellular Ca^2+^ and the activation of its downstream RhoA/LIMK2/cofilin and RhoA/MLCK signaling pathways, which maintained HUASMCs in a state of excessive-contraction. With inhibition of Cx43-GJs by propofol in Ang II-pretreated HUASMCs, intracellular Ca^2+^ and its downstream signaling pathways were dramatically inhibited, which ultimately excessively relaxed HUASMCs. This is the reason why the blood pressure fluctuation of patients with chronic hypertension was more severe after receiving propofol induction.

Video Abstract

**Supplementary Information:**

The online version contains supplementary material available at 10.1186/s12964-023-01176-3.

## Introduction

Violent fluctuations in blood pressure are considered to be a vital cause of perioperative cordis and cerebral accidents [1]. Propofol is a common intravenous anesthetic with a strong vasodilative effect, thereby often leading to hypotension during the induction phase of anesthesia [2]. Importantly, this phenomenon is more obvious in patients with chronic hypertension [3]. The development of chronic hypertension could be attributed to the decrease in blood vessel elasticity induced by long-term excessive contraction [4]. Under such a pathophysiological condition, propofol, with its potent vasodilative effect, can switch the state of blood vessels from overcontraction to excessive relaxation [5]. Eventually, patients with chronic hypertension have abrupt fluctuations in blood pressure and usually experience long-term hypotension because of the deterioration of vascular elasticity. This is one of the elemental reasons for adverse cardiovascular events during anesthesia induction, which can endanger the patients' lives [6, 7]. Therefore, it is crucial to study the effect of propofol on hemodynamics in individuals with chronic hypertension and clarify its internal mechanism.

Hypertension is currently one of the most prevalent cardiovascular diseases in the world, affecting nearly one-fifth of the global population [8]. It has been reported that an irreversible rise in peripheral resistance resulting from prolonged vascular smooth muscle cell (VSMC) constriction exists in chronic hypertensive patients [9]. Long-term exposure to angiotensin II (Ang II), the main component of the renin–angiotensin–aldosterone system (RAAS), is the primary reason [4, 10]. Ang II can promote VSMC proliferation, migration, and vascular remodeling, followed by a thickened vascular lumen and decreased elasticity of resistance arteries [11, 12]. Moreover, the elevated intracellular Ca^2+^ concentration ([Ca^2+^]_i_) in VSMCs caused by Ang II has been shown to play an important role in these physiologic effects [13, 14].

In our previous study, gap junctions (GJs) composed of connexin 43 (Cx43-GJs) were found to be pivotal in propofol-regulated expression of contractile proteins [15]. Cx37, Cx40, Cx43 and Cx45 are expressed in the cardiovascular system, among which Cx43 is the most highly expressed [16]. Six Cx43 proteins circling one another can form one hemichannel and two hemi-channels on adjacent cell membranes can be docked together to form one GJ [17]. GJs allow substances with molecular weights less than 1 kDa to transfer efficiently and freely between adjacent cells, which serves as the physiological underpinning for sustaining circulatory tension and coordinating the contraction and relaxation of blood vessels [18]. Ca^2+^, one of the crucial chemicals transmitted between adjacent cells through GJs, is essential for maintaining cell homeostasis and vascular tension [19, 20]. It has been reported that the alteration of GJ function effectively regulates the change in [Ca^2+^]_i_ in VSMCs, then inducing the contraction and relaxation of blood vessels [21]. Therefore, it deserves our in-depth study to determine whether the severe fluctuation of blood pressure in the stage of anesthesia introduction in chronic hypertensive patients is related to the alteration of Cx43-GJ function and the following changes in [Ca^2+^]_i_.

RhoA is a member of the small GTPase family and previous studies have shown that it can mediate VSMC contraction and vascular remodeling [22, 23]. Notably, RhoA can be activated by Ca^2+^ and regulate the relaxation and contraction of VSMCs through dual regulation [23]. Activated RhoA can stimulate LIMK2 and cofilin phosphorylation,inhibit the degradation of the F-actin cytoskeleton and lead to stable cell contraction [15]. RhoA activation also inhibits the function of myosin light chain phosphatase, which is responsible for the dephosphorylation of MLC2, and thus keeps MLC2 phosphorylated [24]. In addition, the interaction between actin and myosin is implicated in vasoconstriction [25, 26]. Importantly, both the RhoA/LIMK2/cofilin and RhoA/MLCK signaling pathways, which are sensitive to changes in [Ca^2+^]_i_, are implicated in vasoconstriction in chronic hypertension models [27, 28].

In summary, given the importance of Cx43-GJs in altering [Ca^2+^]_i_ and propofol's effective regulation of Cx43-GJ function, we hypothesize that in patients with chronic hypertension, once the function of Cx43-GJs in controlling [Ca^2+^]_i_ is disrupted, it might induce abrupt fluctuations in blood pressure during anesthesia induction. This change may provide a new mechanical explanation for the more severe blood pressure fluctuations caused by propofol in chronic hypertensive patients and a target for therapeutic intervention to prevent such swings during anesthesia induction.

## Materials and methods

### Cell culture and treatments

HUASMCs, certified for the smooth muscle cell marker alpha-smooth muscle actin ($\alpha$- SMA) with immunofluorescence, were purchased from Procell (Wuhan, China). Cells were cultured in Dulbecco's modified Eagle’s medium (DMEM, KeyGEN BioTECH, Nanjing, China) with 10% fetal bovine serum (FBS, Invitrogen, Carlsbad, USA) and 1% penicillin–streptomycin (Invitrogen) and grown in a sterilized cell incubator (Thermo Fisher Scientific, Waltham, USA) at 37 °C, 90% humidity, and 5% CO2. Cells between Passage 3 and Passage 8 were used in all experiments in this study.

Cells were serum-starved for 24 h in DMEM before drug treatment, to switch the cell phenotype from proliferating to contractile [29]. Treatment with 1 μM Ang II for 12 h has been found to significantly promote VSMC proliferation and migration, which are indicators of vascular remodeling and are thought to be the main causes of hypertension [30]; hence, 1 μM Ang II (G-CLONE, Beijing, China) was administered to HUASMCs for 12 h. In accordance with an earlier study [15], we pretreated cells with 30 μM propofol (Kelun, Sichuang, China) to explore the changes in VSMCs in patients with hypertension and with propofol administration at the early stage of anesthesia induction. In addition, cells were treated with an intracellular calcium chelator (BAPTA-AM, 10 μM, APExBIO, Houston, USA), the specific Cx43 gap junction agonist retinoic acid (RA, 1 μM, MedChemExpress, New Jersey, USA) [31], a specific Cx43 gap junction blocker (Gap26, 0.25 mg/ml, APExBIO, Houston, USA), and a selective RhoA inhibitor (50 μM, Rhosin hydrochloride, APExBIO).

### F-actin staining assay

Briefly, HUASMCs were plated in 12-well plates until the growth density reached 80% to 90%, followed by the aforementioned drug processing. After that, the cells were rinsed with PBS (Invitrogen, pH 7.4) once, fixed with 4% paraformaldehyde (JTW-003–500, JETWAY, Guangzhou, China) for 20 min, permeabilized with 0.5% Triton X-100 (Solarbio, Beijing, China) for 20 min, and then blocked with 5% BSA in PBS for 30 min at room temperature. Finally, the cell nuclei were stained with 4,6-diamidino-2-phenylindole (DAPI, Servicebio, Wuhan, China) for 4 min at room temperature in the dark after the F-actin had been labeled with FITC-phalloidin (100 nM, Servicebio, Wuhan, China) in PBS for 30 min. Three different fields were captured in every experiment by the fluorescence microscope equipped with a 470 nm emission/525 nm emission filter set, a digital CCD camera and 40 × objective (Thermo Fisher Scientific). The fluorescence intensity was analyzed by ImageJ based on an earlier study [32]. The experiment was repeated 3–5 times (*n* = 3–5).

### Immunofluorescence (IF) staining

The previous steps of immunofluorescence are largely comparable with F-actin staining assay. After the cells were blocked with 5% BSA for 30 min, they were incubated overnight at 4 °C with primary antibodies diluted in PBS, including anti-p-MLC2 (#95777S, Cell Signaling Technology, 1:600), anti-MLC2 (#340279, Proteintech, 1:300), and anti-Cx43 (#10906–1-AP, ZEN-BIOSCIENCE, 1:200). Then, the cells were further incubated with Alexa Fluor 594-conjugated anti-rabbit secondary antibody (#550043, ZEN-BIOSCIENCE,1:300) or Alexa Fluor 488-conjugated anti-rabbit secondary antibody (#4412S, Cell Signaling Technology, 1:300) in PBS for 1 h at room temperature and in the dark before being exposed to DAPI for 3 min. Three different fields were captured in every experiment by a fluorescence microscope equipped with a 470 nm emission/525 nm emission filter set, a digital CCD camera and a 40 × objective (Thermo Fisher Scientific). The procedure for the measurement of fluorescence intensity was similar to that for the measurement of F-actin. The experiment was repeated 3–5 times (*n* = 3–5).

### Intracellular free Ca^2+^ measurement via flow cytometry

HUASMCs were seeded into 6-well cell plates and cultured to at least 90% confluence. Following drug treatment, cells were washed with PBS once. Cells were then centrifuged after being digested with 0.25% trypsin. Then, equal amounts of Hanks balanced salt solution (HBSS) resuspending solution containing Fluo-3/AM (4, Molecular Probes, AAT Bioquest, USA) were added to each centrifugal tube for cultivation in the dark at room temperature for 30 min. The sample will be stained, and then cleaned twice with HBSS before being transferred to the flow tube. Finally, CytoFLEX LX flow cytometry (Beckman, Pasadena, USA) was used to assess the intracellular Ca^2+^ concentration.

### “Parachute” dye-coupling assay

HUASMCs were inoculated into 6-well plates and then cultured to confluence. Donor cells were taken from one of the wells without drug treatment and treated with the fluorescent indicator calcein-AM (Sigma-Aldrich, 25 pM) and Dil (Invitrogen, 2 μg/ml) in DMEM at 37 °C for 30 min in the dark. The donor cells were then centrifuged at 1,000 rpm for 5 min after being washed with PBS three times and digested with 0.25% trypsin. The recipient cells were then injected with the donor cells at a density of 500/well, and the cells were cultured in a sterile cell incubator for 4 h to induce the formation of gap junctions. Theoretically, Dil (red) is unable to transfer between cells and thus is usually used to label the “donor cells”, while Calcein-AM (green) can transfer to adjacent cells by gap junctions. Therefore, the ratio of the number of “recipient cells” (green) and “donor cells” (yellow) was used to judge GJ function based on an earlier study [33]. Images were taken with a fluorescence microscope (Thermo Fisher Scientific). Three different fields were captured in each experiment. The experiment was repeated 3–5 times (*n* = 3–5).

### Western blot

The produced lysate was added to the cells and reacted at 4 °C for 30 min in accordance with the manufacturer's instructions (KeyGEN, Nanjing, China), which resulted in complete cell lysis. After centrifugation at 12,500 rpm at 4 °C for 12 min, the supernatants were transferred to a fresh tube. Proteins concentrations were measured with a BCA Protein Assay Kit (Beyotime Biotechnology, Shanghai, China) by using a spectrophotometer. Samples were blended with loading buffer (Yeasen, Shanghai, China), and boiled for five minutes at 100 °C. Equal amounts of protein samples were separated by 8–12% SDS-PAGE (GeneScript, New Jersey, USA) and then electrotransferred onto polyvinylidene fluoride membranes (Immobilon-PSQ, Merck Millipore, Ltd., Darmstadt, Germany). The membrane was then incubated with the primary antibodies (mentioned below) at 4 °C overnight after being blocked with 5% nonfat milk at room temperature for 1–2 h. Subsequently, the membrane was rinsed with TBST (TBS: Servicebio; Tween 20: BioFroxx, Guangzhou, China), followed by an-hour incubation with the appropriate horseradish peroxidase-conjugated secondary antibodies (#380626, GAPDH, ZEN-BIOSCIENCE, 1:20000). Finally, the results were analyzed by chemiluminescence (Tanon5200, Shanghai, CN) and ImageJ software (National Institutes of Health, Bethesda, USA). The results are presented as values that have been adjusted for GAPDH and the control.

Primary antibodies against Cx43 (#3512S, 1:1000, Cell Signaling Technology, Boston, MA, USA), RhoA (#sc-418, 1:1000, Santa Cruz Biotechnology, Dallas, USA), LIMK2 (#df3211, 1:1000, Affinity Biosciences, Jiangsu, China), phospho-LIMK2 (#ab38499, 1:1000, Abcam), cofilin (#7416354, 1:1000, Affinity Biosciences), phospho-Cofilin (#SN8071401, 1:1000, Affinity Biosciences), and MLCK (#381015, 1:1000, ZEN-BIOSCIENCE) were used.

### siRNA transfection

Cx43-siRNA (CAGUCUGCCUUUCGUUGUA, RiboBio, Guangzhou, CN) was applied to modify the communicating function of Cx43-GJs [15]. The control group was given a sequence-scrambled siRNA as a negative control (NC-siRNA) for comparison. Following the manufacturer's instructions, siRNA transfection was performed using a transfection kit (RiboBio).

### Statistical analysis

All results are reported as the mean ± standard error (SEM). All experiments were repeated at least three times in biological replicates. Data were normalized to those of the control group to decrease the discrepancy brought by techniques. Statistical analysis was performed using GraphPad Prism 8 software (GraphPad Software, San Diego, USA). Multiple comparisons among groups were performed using one-way ANOVA, followed by Tukey post hoc comparisons. Statistical significance was set at a *p*-value of < 0.05.

## Results

### Propofol induced excessive relaxation in Ang II- pretreated HUASMCs compared to normal HUASMCs

**Fig. 1:**
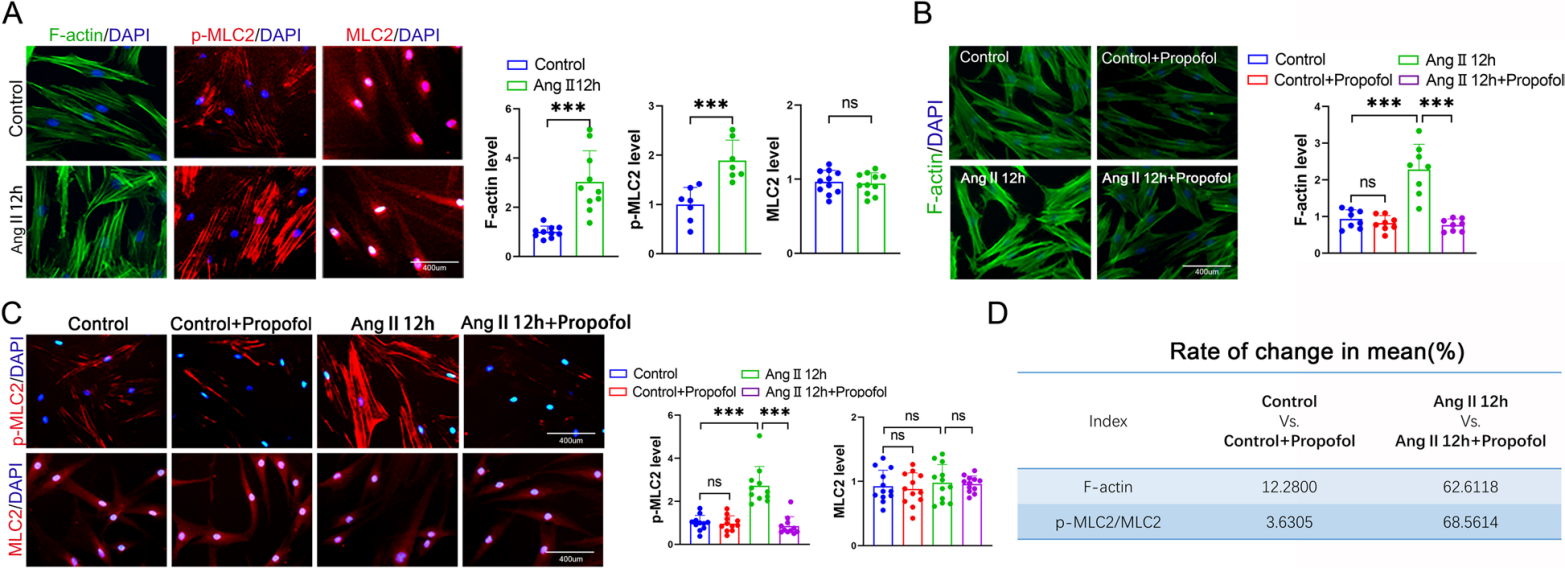
Propofol induced excessive relaxation in Ang II-pretreated HUASMCs compared to normal HUASMCs. **A** F-actin was stained by FITC-phalloidin (green), p-MLC2 and MLC2 were labeled by cellular immunofluorescence (red) and cell nuclei were stained by DAPI (blue) in HUASMCs. Each dot represents the fluorescence intensity of one field. Three different fields were captured in every experiment. The experiment was repeated 3–5 times (*n* = 3–5 per group). **B** F-actin was stained with FITC-phalloidin (green) and cell nuclei was stained with DAPI (blue) in HUASMCs. Each dot represents the fluorescence intensity of one field. Three different fields were captured in every experiment. The experiment was repeated 3 times (*n* = 3 per group). **C** p-MLC2 and MLC2 were labeled by cellular immunofluorescence (red) and cell nuclei were stained by DAPI (blue) in HUASMCs. Each dot represents the fluorescence intensity of one field. Three different fields were captured in every experiment. The experiment was repeated 3–5 times (*n* = 3–5 per group). **D** The change rate of F-actin and p-MLC2/MLC2 in mean between Control vs. Control + propofol and Ang II vs. Ang II + propofol. *p < 0.05, ** p < 0.01, *** *p* < 0.001. All values expressed as mean ± SEM

F-actin polymerization and MLC2 phosphorylation are key contractile elements in controlling cell contraction and relaxation in smooth muscle cells [25]. Ang II has been proven to be a pivotal product mediating smooth muscle cell contraction and can even result in the development of hypertension [12, 30]. Therefore, we used Ang II (1 μM, 12 h) to pretreat HUASMCs, and observed that both F-actin polymerization and MLC2 phosphorylation were significantly enhanced (Fig. 1A). However, this positive regulation could be substantially reversed by propofol (Fig. 1B and C), a well-known anesthetic characterized by hypotensive response. Moreover, the dilative effect exerted by propofol on Ang II-pretreated HUASMCs was more obvious than that of normal HUASMCs, manifested as a lager magnitude of decline in F-actin polymerization (62.6118% *vs.* 12.28%) and MLC2 phosphorylation (68.5614% *vs.* 3.6305%) (Fig. 1D). These results suggest that propofol tends to trigger excessive relaxation in Ang II-pretreated HUASMCs, compared with normal HUASMCs.

### Propofol induced a greater magnitude of [Ca^2+^]_i_ decline in Ang II-pretreated HUASMCs than normal HUASMCs

**Fig. 2:**
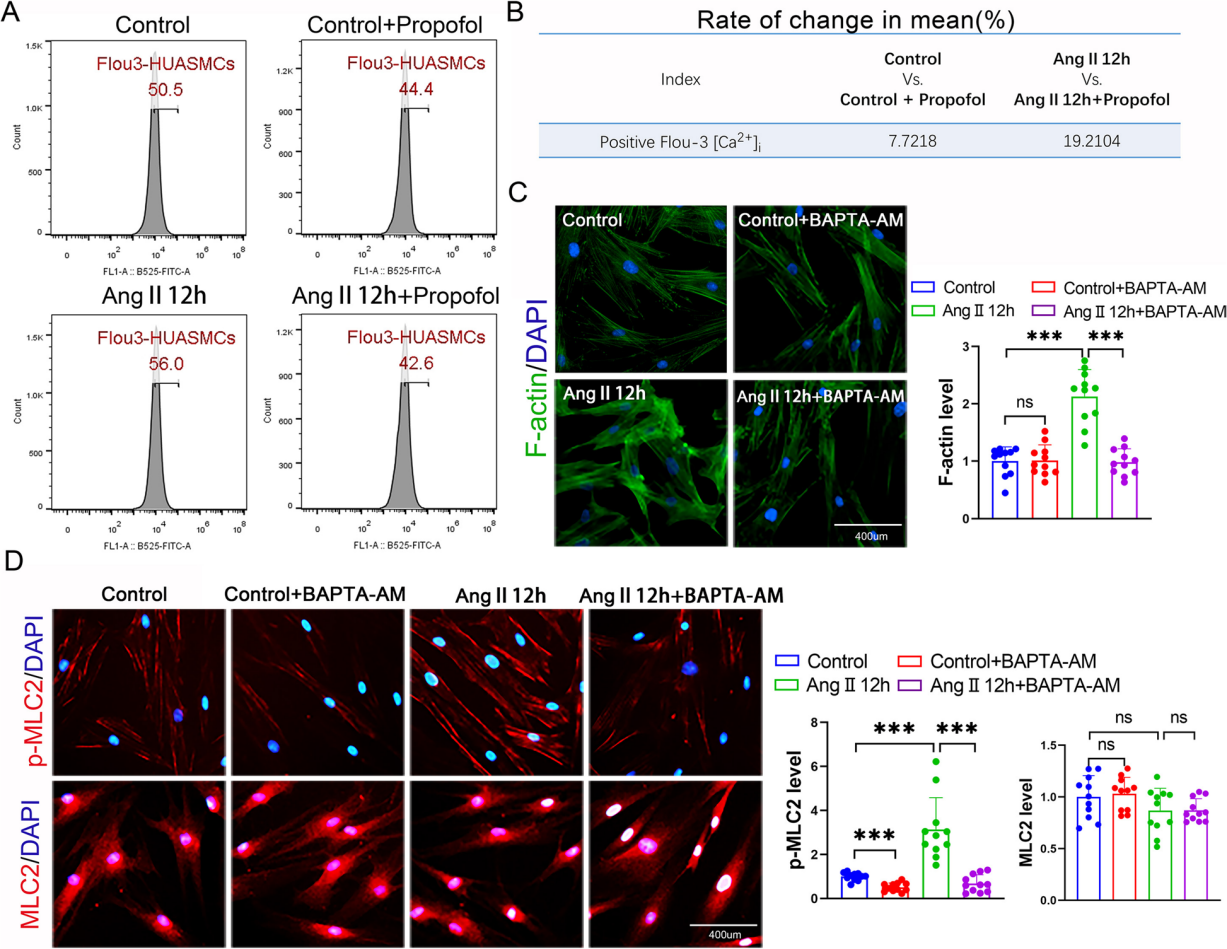
Propofol induced a greater magnitude of [Ca^2+^]_i_ decline in Ang II-pretreated HUASMCs. **A** Detection of [Ca^2+^]_i_ with fluo3-AM by flow cytometry on HUASMCs. **B** The change rate of [Ca^2+^]_i_ in mean between Control vs. Control + propofol and Ang II vs. Ang II + propofol. **C** F-actin was stained with FITC-phalloidin (green) and cell nuclei was stained with DAPI (blue) in HUASMCs. Each dot represents the fluorescence intensity of one field. Three different fields were captured in every experiment. The experiment was repeated 4 times (*n* = 4 per group). **D** p-MLC2 and MLC2 were labeled by cellular immunofluorescence (red) and cell nuclei were stained by DAPI (blue) in HUASMCs. Each dot represents the fluorescence intensity of one field. Three different fields were captured in every experiment. The experiment was repeated 4 times (*n* = 4 per group). *p < 0.05, ** *p* < 0.01, *** *p* < 0.001. All values expressed as mean ± SEM

As reported, increased accumulation of intracellular Ca^2+^ plays an important role in stimulating cell contraction [13, 14]. In this study, we used a Fluo-3 probe to determine the change in [Ca^2+^]_i_ and found that propofol could apparently decrease [Ca^2+^]_i_ in HUASMCs with or without Ang II exposure (Fig. 2A), but in the Ang II- pretreated HUASMCs group, [Ca^2+^]_i_ had a larger magnitude of decline (19.2104% vs. 7.7218%) (Fig. 2B), which was highly consistent with the alteration in F-actin polymerization and MLC2 phosphorylation. With the decrease in [Ca^2+^]_i_ in HUASMCs induced by BAPTA-AM, Ang II-induced F-actin polymerization and MLC2 phosphorylation were also significantly attenuated (Fig. 2C and D). These results underlined a potential mechanism by which propofol-induced excessive dilation in Ang II-pretreated HUASMCs may be correlated with the decreased magnitude of [Ca^2+^]_i_.

### Propofol attenuated Ca^2+^ transfer between neighboring HUASMCs, F-actin polymerization and MLC2 phosphorylation by inhibiting the function of Cx43-GJs

**Fig. 3:**
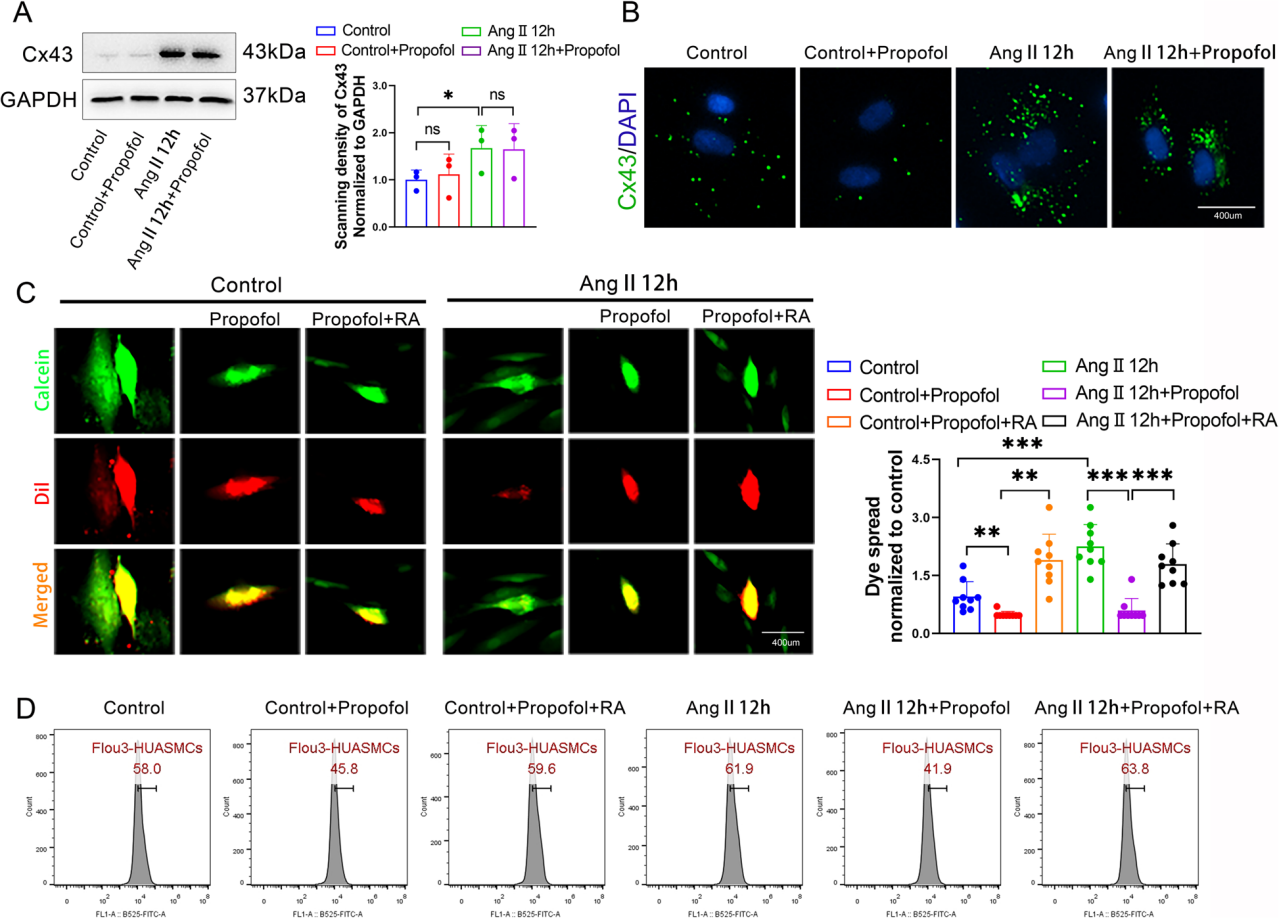
Propofol attenuated Ca^2+^ transfer between neighboring HUASMCs by inhibiting the function of Cx43-GJs. **A**, **B** Western blot (**A**) and cell immunofluorescence (**B**) were used to test Cx43 expression. Cx43 protein in (**B**) was stained in green and cell nuclei were stained in blue. GAPDH served as a loading control. *n* = 3 per group. **C** “Parachute” dye coupling assay was applied to measure the function of GJs. In merged images, donor cells were stained in yellow by Calcein (green) and Dil (red); receiver cells were stained in green with Calcein. Each dot represents the ratio of “recipient cells” (green)/ “donor cells” (yellow) in one field. Three different fields were captured in every experiment. The experiment was repeated 3 times (*n* = 3 per group). **D** Detection of [Ca^2+^]_i_ with fluo3-AM by flow cytometry on HUASMCs. *p < 0.05, ** *p* < 0.01, *** *p* < 0.001. All values expressed as mean ± SEM

**Fig. 4:**
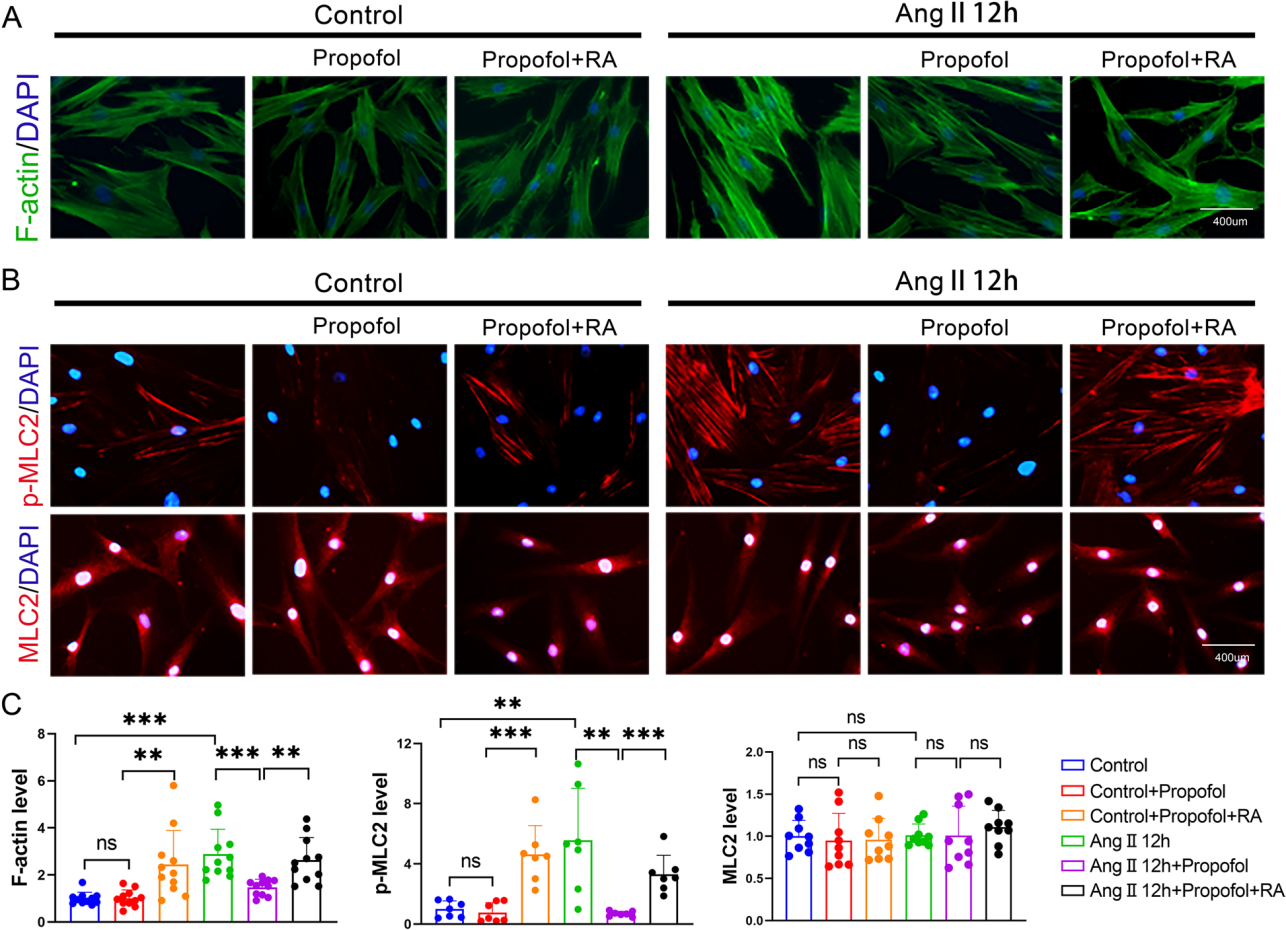
Enhancing Cx43-GJs function with retinoic acid reversed propofol-induced excessive relaxation in Ang II-pretreated HUASMCs. **A** F-actin was stained with FITC-phalloidin (green) and cell nuclei was stained with DAPI (blue) in HUASMCs. **B** p-MLC2 and MLC2 were labeled by cellular immunofluorescence (red) and cell nuclei were stained by DAPI (blue) in HUASMCs. **C** Bar plots showed the statistical results of F-actin level, p-MLC2 level and MLC2 level. Each dot represents the fluorescence intensity of one field. Three different fields were captured in every experiment. The experiment was repeated 3–5 times (*n* = 3–5 per group). ** *p* < 0.01, *** *p* < 0.001. All values expressed as mean ± SEM

Cx43-GJs can act as Ca^2+^ channels for transferring Ca^2+^ between neighboring VSMCs, efficiently regulating [Ca^2+^]_i_, which constitutes the biological basis of the synchronization of vascular smooth muscle contraction [18, 34]. Therefore, we first detected the changes in Cx43 expression in HUASMCs and the results showed that Cx43 expression could be upregulated by Ang II but was not downregulated by propofol (Fig. 3A and B). Considering that propofol attenuated [Ca^2+^]_i_ in HUASMCs as shown in Fig. 2, we mainly observed the effects of Cx43-GJ function on HUAMSC contraction and relaxation in subsequent experiments. Figure 3C demonstrates that the function of Cx43-GJs could be enhanced by Ang II pretreatment, and this effect was reversed by propofol. Of note, the magnitude of the decline in Cx43-GJ function caused by propofol was more significant in the Ang II pretreatment group. The effects of propofol could be antagonized by RA, an enhancer of Cx43-GJs, in both the control groups and Ang II pretreatment groups. With the alteration of Cx43-GJ function, [Ca^2+^]_i_ (Fig. 3D), F-actin polymerization and MLC2 phosphorylation (Fig. 4A-C) also showed corresponding changes. These indicators were all enhanced by Ang II pretreatment, but decreased by propofol. Moreover, RA antagonized the effect of propofol. We should also note that in the Ang II pretreatment group, the magnitude of decline caused by propofol was more obvious. This phenomenon might be the underlying mechanism of propofol-induced excessive relaxation in Ang II -HUASMCs.

### Inhibition of Cx43-GJs with specific Gap26 and siRNA attenuated Ca^2+^ transfer between neighboring HUASMCs, F-actin polymerization and MLC2 phosphorylation

**Fig. 5:**
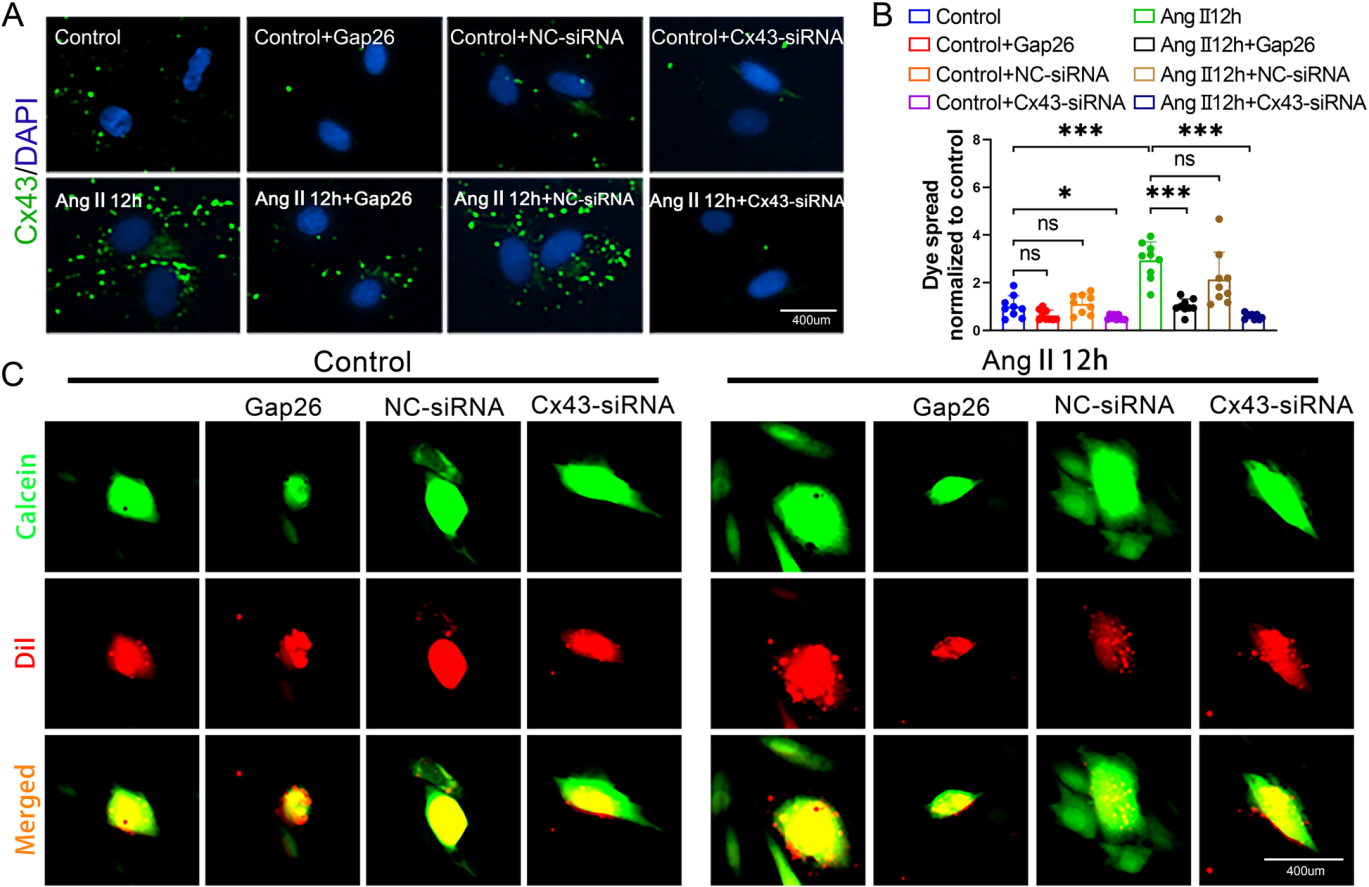
Gap26 and Cx43-siRNA significantly inhibited Cx43-GJs function in Ang II-pretreated HUASMCs. **A** Cell immunofluorescence was used to test Cx43 expression. Cx43 protein in was stained in green and cell nuclei were stained in blue. *n* = 3–5 per group. **B**, **C** “Parachute” dye coupling assay was applied to measure the function of GJs. In merged images, donor cells were stained in yellow by Calcein (green) and Dil (red); receiver cells were stained in green with Calcein. Each dot represents the ratio of “recipient cells” (green)/ “donor cells” (yellow) in one field. Three different fields were captured in every experiment. The experiment was repeated 3 times (*n* = 3 per group). *p < 0.05, *** *p* < 0.001. All values expressed as mean ± SEM

**Fig. 6:**
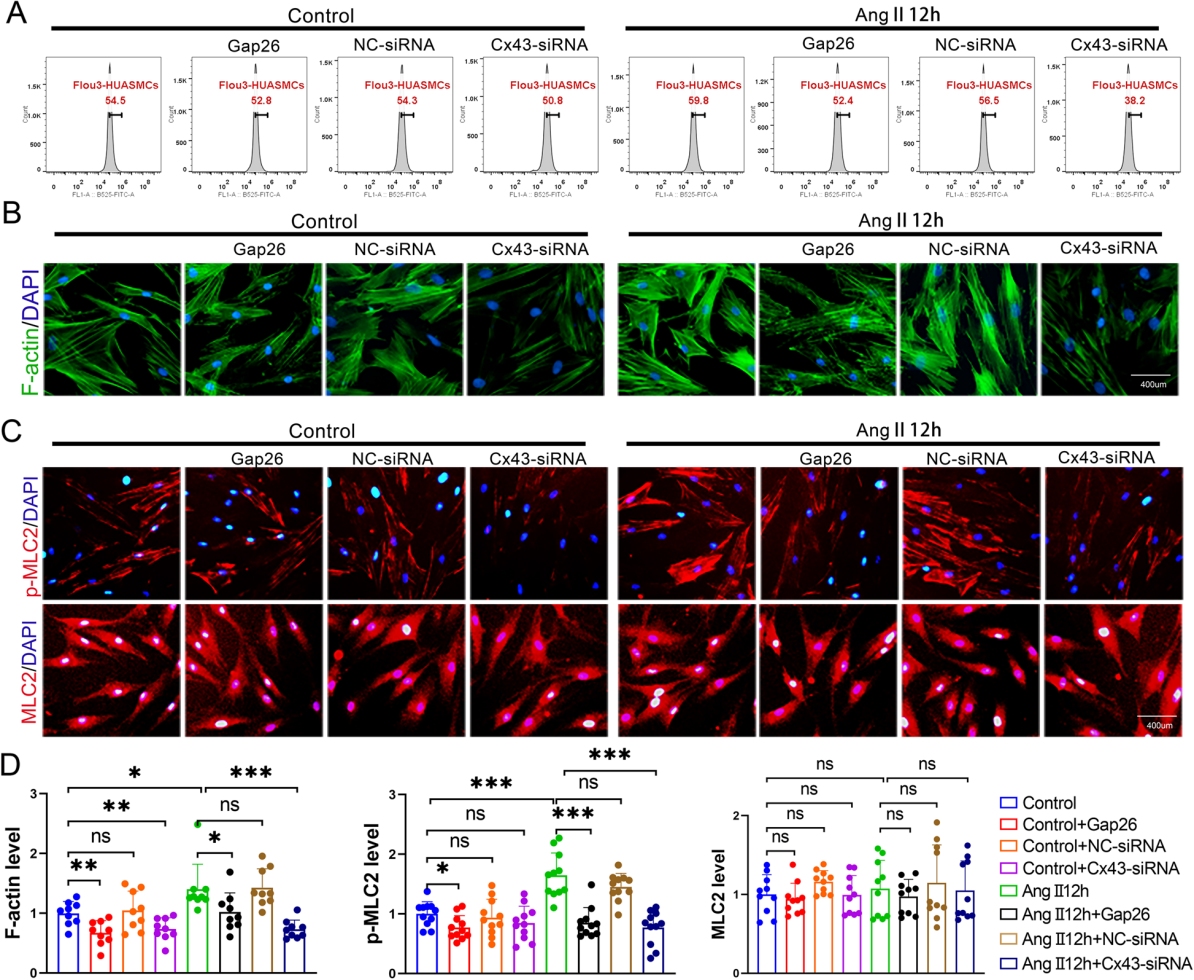
Inhibition of Cx43-GJs function attenuated Ang II-increased [Ca^2+^]_i_, F-actin polymerization and MLC2 phosphorylation. **A** Detection of [Ca^2+^]_i_ with fluo3-AM by flow cytometry on HUASMCs. **B** F-actin was stained with FITC-phalloidin (green) and cell nuclei was stained with DAPI (blue) in HUASMCs. Each dot represents the fluorescence intensity of one field. Three different fields were captured in every experiment. The experiment was repeated 3 times (*n* = 3 per group). **C** p-MLC2 and MLC2 were labeled by cellular immunofluorescence (red) and cell nuclei were stained by DAPI (blue) in HUASMCs. Each dot represents the fluorescence intensity of one field. Three different fields were captured in every experiment. The experiment was repeated 3–4 times (*n* = 3–4 per group). **D** Quantitation of fluorescence intensity (**B**, **C**) in each group. *p < 0.05, ** *p* < 0.01, *** *p* < 0.001. All values expressed as mean ± SEM

To confirm the hypothesis that we have mentioned above and further clarify the function of Cx43-GJs in propofol-induced excessive relaxation of HUASMCs, we used Gap26 and siRNA targeting Cx43 to specifically alter the function of Cx43-GJs, both of which decreased the effect of Cx43-GJs on HUASMCs, especially in the Ang-II pretreated group (Fig. 5A-C). As expected, with the decrease in Cx43-GJ function following the use of Gap26 or Cx43-siRNA, [Ca^2+^]_i_ was obviously diminished in Ang II-pretreated HUASMCs (Fig. 6A). Moreover, compared with that of the control group, the magnitude of decline in F-actin polymerization and MLC2 phosphorylation was much more significant in the Ang II-pretreated group (Fig. 6B-D). In summary, these results demonstrated that Cx43-GJ-mediated changes in [Ca^2+^]_i_ may be crucial in propofol-induced excessive relaxation in HUASMCs, especially in the Ang II-pretreated group.

### Downregulation RhoA expression contributed to propofol-induced excessive relaxation of Ang II-pretreated HUASMCs

**Fig. 7:**
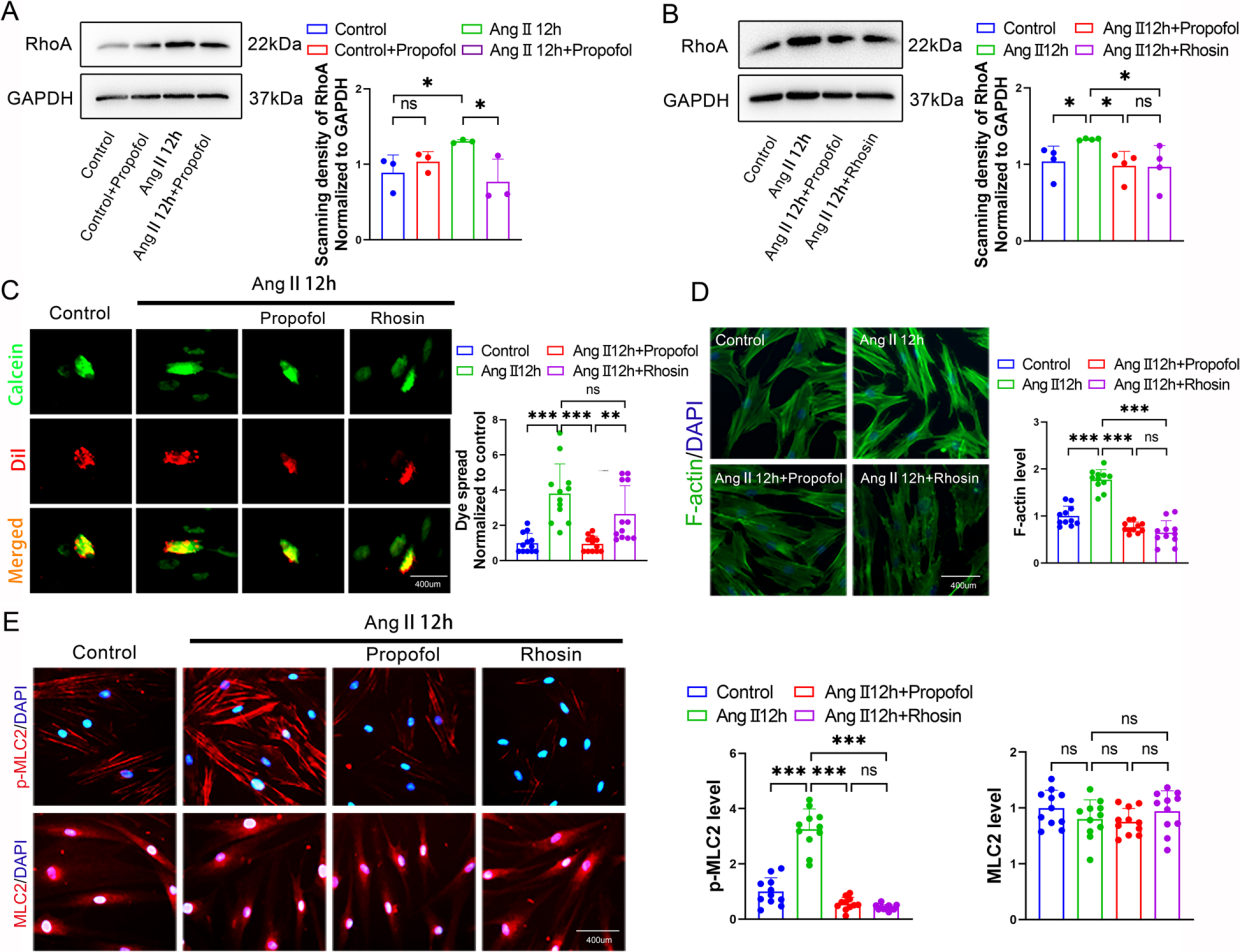
Down-regulated RhoA expression contributed to propofol-induced excessive relaxation in Ang II-pretreated HUASMCs. **A**, **B** western blot was used to test RhoA expression. GAPDH served as a loading control. *n* = 3 per group in (**A**) and *n* = 4 per group in (**B**). **C** “Parachute” dye coupling assay was applied to measure the function of GJs. In merged images, donor cells were stained in yellow by Calcein (green) and Dil (red); receiver cells were stained in green with Calcein. Each dot represents the ratio of “recipient cells” (green)/ “donor cells” (yellow) in one field. Three different fields were captured in every experiment. The experiment was repeated 4 times (*n* = 4 per group). **D** F-actin was stained with FITC-phalloidin (green) and cell nuclei was stained with DAPI (blue) in HUASMCs. Each dot represents the fluorescence intensity of one field. Three different fields were captured in every experiment. The experiment was repeated 4 times (*n* = 4 per group). **E** p-MLC2 and MLC2 were labeled by cellular immunofluorescence (red) and cell nuclei were stained by DAPI (blue) in HUASMCs. Each dot represents the fluorescence intensity of one field. Three different fields were captured in every experiment. The experiment was repeated 4 times (*n* = 4 per group). *p < 0.05, ** *p* < 0.01, *** *p* < 0.001. All values expressed as mean ± SEM

According to different reports, the function of RhoA in regulating cell contraction and relaxation is closely related to [Ca^2+^]_i_ [23, 26]. Figure 7A shows that the Ang II-induced increase in RhoA expression could be reversed by propofol, but this effect was not observed in the control group. Notably, the inhibitory effect of propofol on RhoA was almost the same as that of the RhoA-specific inhibitor, Rhosin (Fig. 7B). However, Rhosin had no effect on Cx43-GJ function in the Ang II-pretreated HUASMC group (Fig. 7C). With the inhibition of RhoA by Rhosin and propofol, F-actin polymerization and MLC2 phosphorylation in Ang II-HUASMCs were significantly inhibited (Fig. 7D and E). In the case of no effect on Cx43-GJ function, the effect of Rhosin on contractile proteins is similar to that of propofol.

### Propofol inhibited the activation of the RhoA/LIMK2/cofilin and RhoA/MLCK signaling pathways

**Fig. 8:**
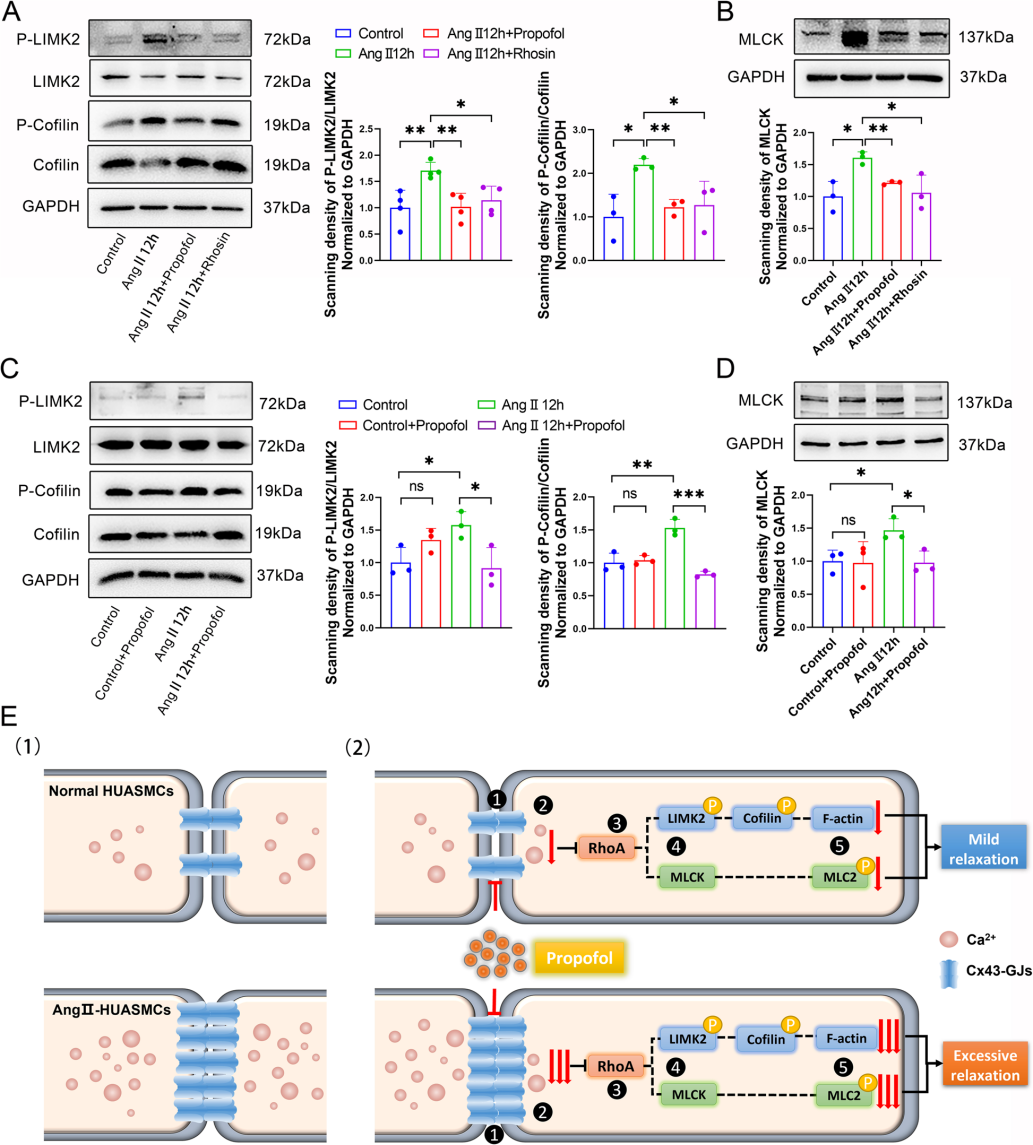
Propofol inhibited the activation of RhoA/LIMK2/cofilin and RhoA/MLCK signaling pathways. **A** Levels of P-LIMK2, LIMK2, P-cofilin and cofilin on HUASMCs were tested by western blot. GAPDH served as a loading control. *n* = 4 per group. **B** Levels of MLCK on HUASMCs were tested by western blot. GAPDH served as a loading control. *n* = 3 per group. **C** Levels of p-LIMK2, LIMK2, p-cofilin and cofilin on HUASMCs were tested by western blot. GAPDH served as a loading control. *n* = 3 per group. **D** Levels of MLCK on HUASMCs were tested by western blot. GAPDH served as a loading control. *n* = 3 per group. *p < 0.05, ** *p* < 0.01, *** *p* < 0.001. All values expressed as mean ± SEM. **E** The scheme of the mechanism that propofol induces excessive relaxation in Ang II-HUASMCs. (1) Comparison of the number and function of Cx43-GJs and [Ca^2+^]_i_ in normal and Ang II-HUASMCs. (2) ① When HUASMCs are treated with propofol, propofol causes a prominent decrease in the function of Cx43-GJs in Ang II-HUASMCs compared with normal HUASMCs; ② This results in a more substantial decrease of intracellular Ca^2+^ mobility in Ang II-HUASMCs; ③-④ Subsequently, propofol also leads to a stronger inhibition on the activation of RhoA protein and its downstream- RhoA/LIMK2/cofilin and RhoA/MLCK signaling pathways in Ang II-HUASMCs; ⑤ Ultimately, compared to normal HUASMCs, the activation of F-actin polymerization and MLC2 phosphorylation were both mitigated more dramatically in Ang II-HUASMCs, which caused excessive relaxation

As reported, both the RhoA/LIMK2/cofolin and RhoA/MLCK pathways can lead to cell contraction [15, 24]. Our results demonstrated that Ang II exposure significantly activated the RhoA/LIMK2/cofilin and RhoA/MLCK signaling pathways, manifested as an increase in key molecules, such as p-LIMK2, p-cofilin and MLCK, which was reversed by propofol and Rhosin (Fig. 8A and B). Importantly, the inhibitory effects of propofol on the RhoA/LIMK2/Cofilin and RhoA/MLCK signaling pathways were much more significant in the Ang II-pretreated group than in the control group (Fig. 8C and D).

## Discussion

Compared to healthy subjects, chronic hypertensive patients are prone to have a higher rate of hypotension events and experience a deeper drop in blood pressure after receiving propofol induction [3], which may threaten the life of these patients. This phenomenon deserves close attention from anesthesiologists. According to different reports, Ang II has been proven to be a pivotal product mediating smooth muscle cell contraction and even resulting in the development of hypertension [12–14]. Thus, we built a smooth muscle cell contraction model in vitro by pretreating HUASMCs with Ang II to explore the underlying mechanism by which propofol induces a greater decrease in blood pressure in patients with chronic hypertension. Our findings showed that long-term Ang II could induce Cx43 expression in HUASMCs, which mediated substantial Ca^2+^ transfer between neighboring cells. Then, the Ca^2+^-related protein, RhoA, and its downstream-RhoA/LIMK2/cofilin and RhoA/MLCK signaling pathways were both activated, inducing contractile proteins, such as F-actin polymerization and MLC2 phosphorylation, which were markedly upregulated. This effect could be reversed by propofol, the mechanism of which was that propofol efficiently inhibited the Ca^2+^ transfer mediated by Cx43-GJs. Of note, propofol pretreatment resulted in a more drastic reduction in Ca^2+^ transfer between neighboring Ang II-pretreated HUASMCs by inhibiting Cx43-GJ function, which caused excessive HUASMCs relaxation, compared with that of the normal HUASMCs (Fig. 8E). These results explained why the blood pressure fluctuation of patients with chronic hypertension was more severe after receiving propofol induction.

Ang II is a crucial material basis in the development of essential hypertension. This molecule can alter the state of VSMC contractile proteins, such as F-actin polymerization and MLC2 phosphorylation, which leads to vasoconstriction and blood pressure elevation [28, 35]. It has been reported that Cx43 expression and the function of Cx43-GJs are significantly enhanced in spontaneously hypertensive rats [36]. In the present study, we also confirmed this phenomenon in *vivo*. In addition, we observed that Cx43 expression induced by long-term Ang II in HUASMCs mediated Ca^2+^ transfer between neighboring HUASMCs via Cx43-GJs, along with activation of the downstream RhoA/LIMK2/cofilin and RhoA/MLCK signaling pathways, resulting in excessive HUASMCs contraction. We believe that Cx43 might be a new target to address chronic hypertension. Although our previous study showed that short-term Ang II exposure had no effect on Cx43 expression in HUASMCs, it indeed increased GJ function. Ca^2+^ transfer between neighboring HUASMCs and contractile proteins were both increased. When Ang II was removed, GJ function recovered, and contractile protein also returned to normal levels, which might be the underlying mechanism of white coat hypertension. These results once again confirmed the importance of Cx43 in regulating blood pressure changes. However, of note, the change in Cx43-GJs was different under long-term and short-term Ang II exposure. Long-term Ang II exposure increased the function of Cx43-GJs by upregulating Cx43 expression in HUASMCs, which could not be reversed, while short-term Ang II exposure temporarily altered Cx43-GJ function without affecting Cx43 expression. This result also explained why the blood vessels of patients with chronic hypertension were always in a contractile state. Therefore, propofol, which has a strong inhibitory effect on the function of Cx43-GJs [37], results in a drastic decrease in Ca^2+^ transfer between neighboring HUASMCs and in the expression of contractile proteins, finally leading to excessive relaxation of blood vessels and severe fluctuation in blood pressure.

Hypotension caused by propofol in the process of anesthesia induction is very common and requires the attention of anesthesiologists. Especially for patients with chronic hypertension, the antihypertensive effect of propofol is more significant, which may endanger the life of patients [38]. Cx37, Cx40, Cx43 and Cx45 are expressed in VSMCs. GJs composed of connexins allow free and efficient transfer of substances with molecular weights less than 1 kDa between adjacent cells, such as Ca^2+^. Furthermore, an earlier study demonstrated that GJs could transfer hypercontracture to adjacent cells but that transmission could be largely reversed and the vascular tone was greatly decreased when GJ uncoupler heptanol was applied [39]. These results indicated that GJs may be the essential biological basis for coordinating the synchronous contraction and relaxation of VSMCs. Only by coordinating the synchronous contraction of adjacent VSMCs can vascular smooth muscle truly constrict. Therefore, the number of GJs is critical for vasoconstriction and relaxation. In addition, the expression of Cx43 is the most abundant in VSMCs and has been reported to be remarkably increased in hypertensive patients [36]. Moreover, our previous studies have confirmed that propofol has a strong inhibitory effect on Cx43-GJs, even if the exposure is very short [15]. Thus, we investigated whether the unequal alteration of Cx43-GJ function underlies the mechanism by which patients with chronic hypertension suffered severe fluctuations in blood pressure after using propofol compared to healthy patients. In our recent study, we found that with overinhibition of Cx43-GJ function by propofol, Ca^2+^ transfer would be suppressed dramatically compared with that of patients without hypertension, which may promote patient's blood vessel to change from excessive contraction to excessive relaxation, finally manifesting as severe fluctuation of blood pressure.

Ca^2+^ is acknowledged to play a crucial role in the development of hypertension [40, 41]. Our present study confirmed that Cx43-GJs could regulate [Ca^2+^]_i_ by mediating Ca^2+^ transfer between neighboring HUASMCs, which effectively adjusts RhoA. RhoA is a member of the small GTPase family, and is involved in mediating VSMC contraction, phenotype switching and vascular remodeling [22, 23]. Sensitizing RhoA could activate the RhoA/LIMK2/cofilin and RhoA/MLCK signaling pathways, stimulating F-actin polymerization and MLC2 phosphorylation, which eventually induces cell contraction [23, 28]. In our present study, we demonstrated that Rhosin, a specific RhoA inhibitor, attenuated F-actin polymerization and MLC2 phosphorylation by inhibiting the RhoA/LIMK2/cofilin and RhoA/MLCK signaling pathways, but had no effect on Cx43 expression. Propofol exposure also had the same effects as Rhosin, but the mechanism was different. Propofol inhibited Cx43-GJs and reduced Ca^2+^ transfer; thus Ca^2+^-related RhoA protein and its downstream RhoA/LIMK2/cofilin and RhoA/MLCK signaling pathways were depressed.

To summarize, we first reported that the blood pressure fluctuation of patients with chronic hypertension was more severe after receiving propofol induction and found that Cx43 might play an important part in this process. For patients with chronic hypertension, long-term Ang II exposure resulted in a significant increase in Cx43 expression and the function of Cx43-GJs, which mediated substantial Ca^2+^ transfer between neighboring HUASMCs, compared with the patients without chronic hypertension. Therefore, the patient's blood vessels were in a state of excessive contraction. The inhibitory effect of propofol on Cx43-GJs was very significant and effective. Ca^2+^ transfer was attenuated drastically between neighboring HUASMCs, leading to the patient's blood vessels changing from excessive contraction to excessive relaxation. Finally, we observed that propofol caused more severe blood pressure fluctuations in patients with chronic hypertension during anesthesia induction. However, further *in-vivo* studies are needed to confirm this finding.

## Supplementary Information

**Additional file 1.**

## Data Availability

The authors confirm that the data supporting the findings of this study are available within the article.
