# Supplementary material for: Downregulation of connexin 43-based gap junctions underlies propofol-induced excessive relaxation in hypertensive vascular smooth muscle cells
Source: Cell Commun Signal. 2023 Jun 28;21:163. doi: 10.1186/s12964-023-01176-3 (PMC10304740; doi:10.1186/s12964-023-01176-3)

**Down-regulation of connexin 43 based gap junction underlies propofol-induced excessive relaxation in hypertensive vascular smooth muscle cells**

**Content**

[Figure 3 2](#_Toc127735634)

[Fig. 3A 2](#_Toc127735635)

[Figure 7 3](#_Toc127735636)

[Fig. 7A 3](#_Toc127735637)

[Fig. 7B 3](#_Toc127735638)

[Figure 8 4](#_Toc127735639)

[Fig. 8A 4](#_Toc127735640)

[Fig. 8B 5](#_Toc127735641)

[Fig. 8C 5](#_Toc127735642)

[Fig. 8D 6](#_Toc127735643)

# Figure 3

## Fig. 3A


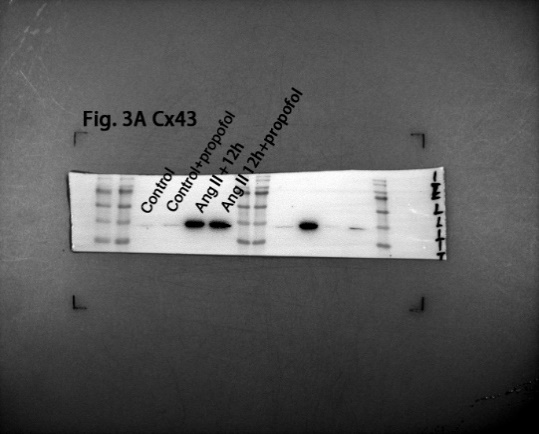

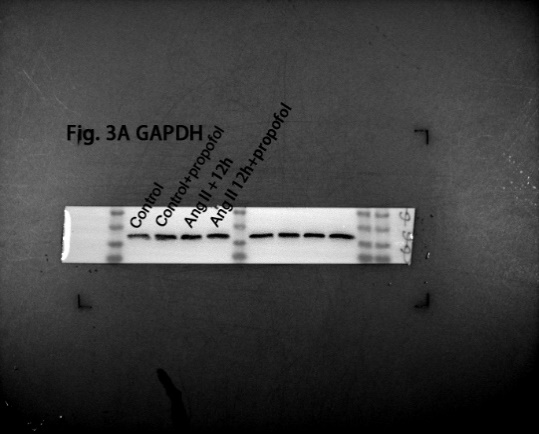


# Figure 7

## Fig. 7A


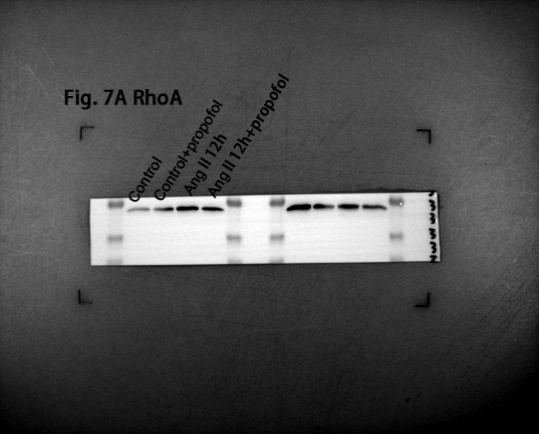

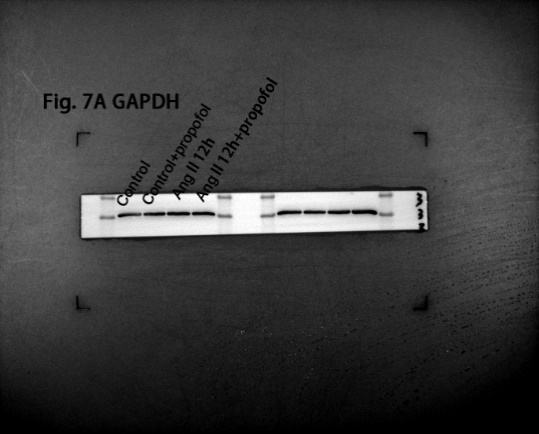


## Fig. 7B


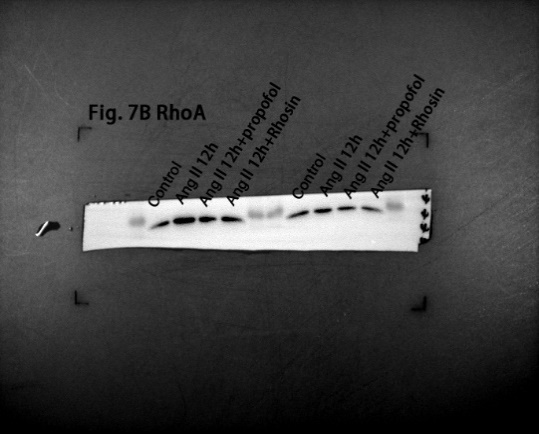

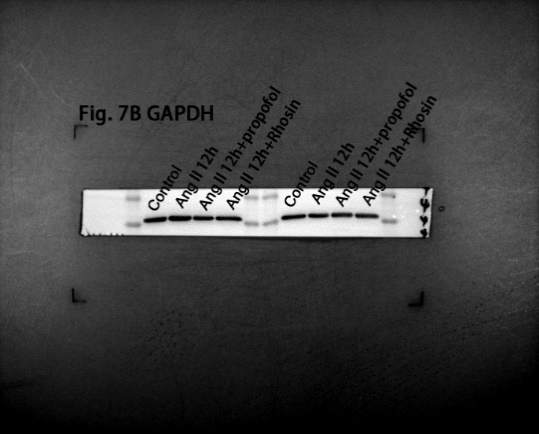


# Figure 8

## Fig. 8A


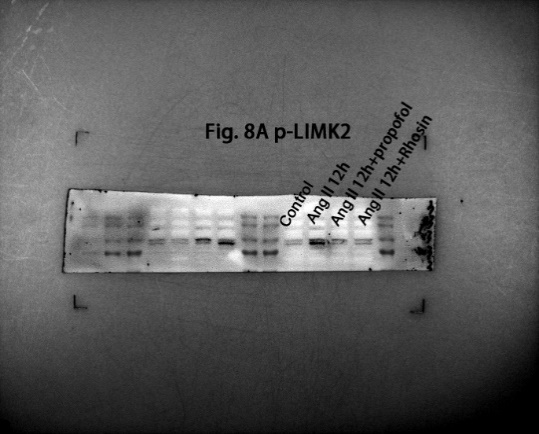

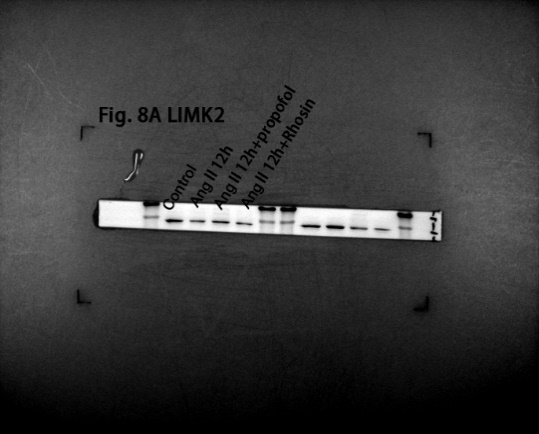


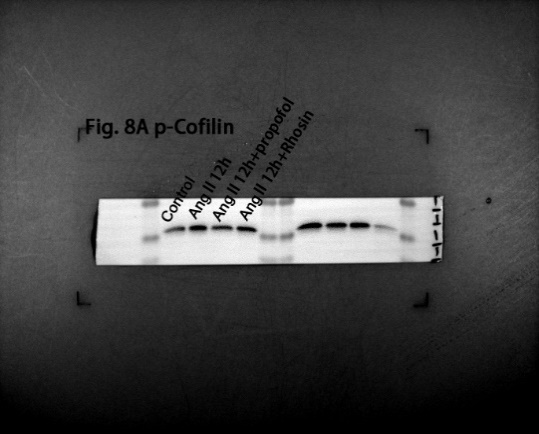

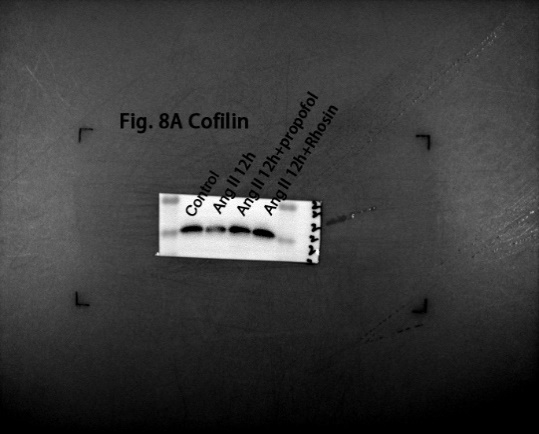


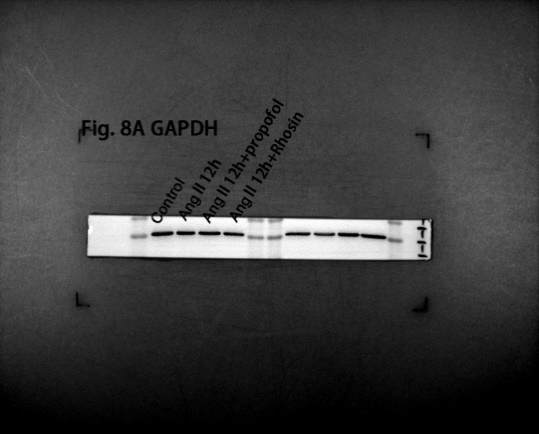


## Fig. 8B


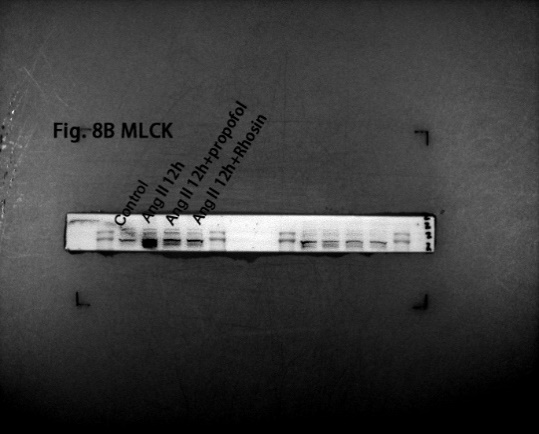

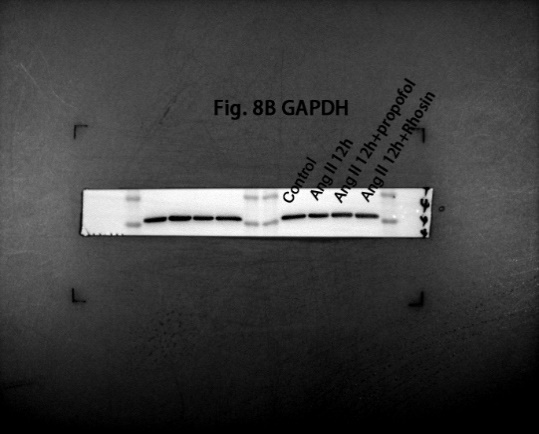


## Fig. 8C


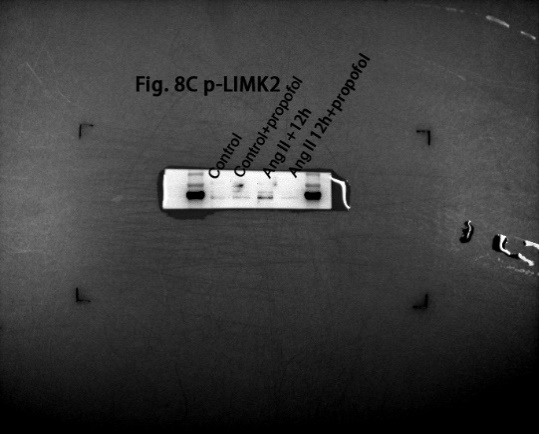

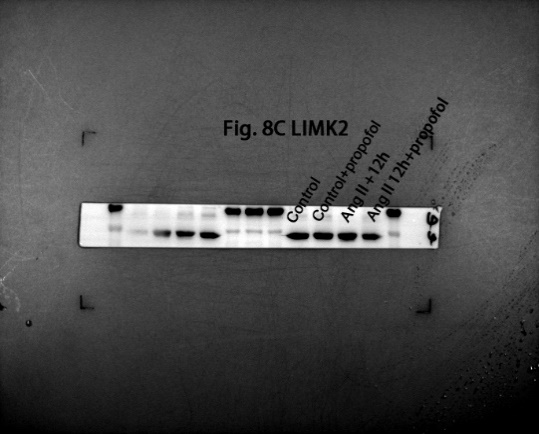


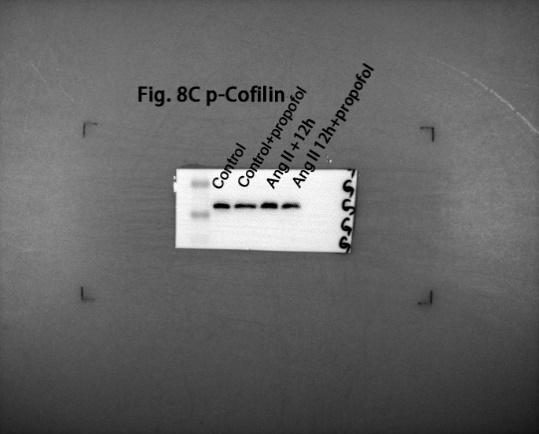

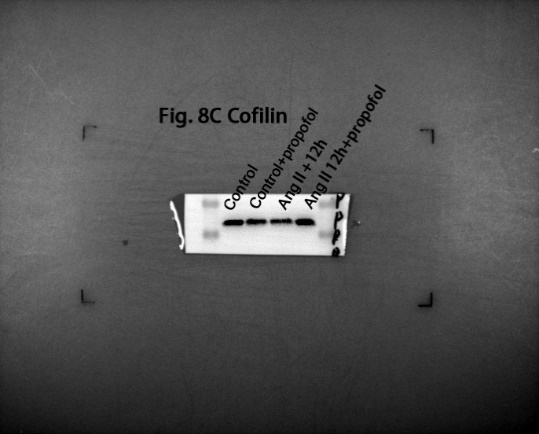


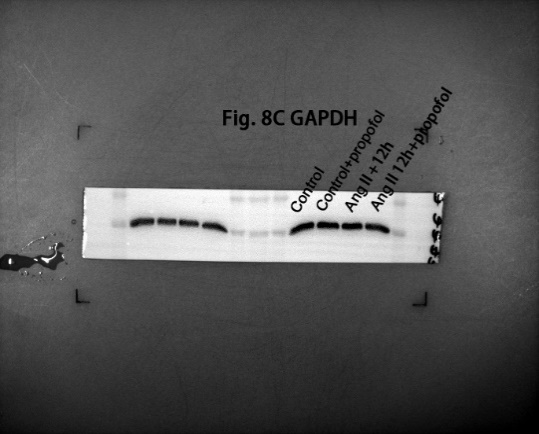


## Fig. 8D


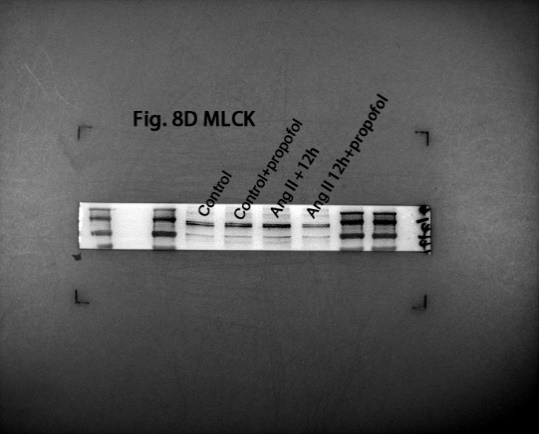

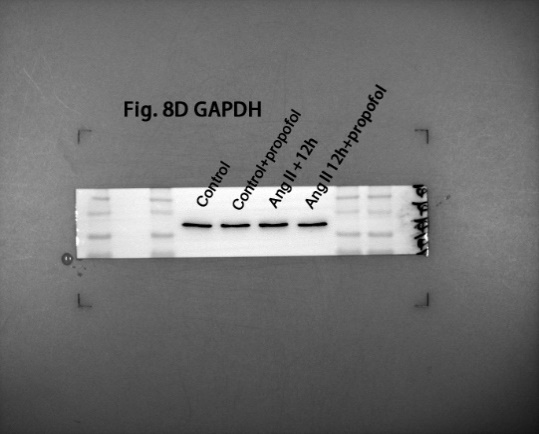

Supplement: Supplementary file 2 — Additional file 1. [file 12964_2023_1176_MOESM1_ESM.docx]
